# Comparison of Croup Management Patterns between Pediatricians and Emergency Medicine Physicians: A Single Pediatric Emergency Department Study

**DOI:** 10.3390/jcm13206095

**Published:** 2024-10-12

**Authors:** Ho-Young Song, Jae-Hyun Kwon, Soo Hyun Park, Min-Jung Kim, Young-Hoon Byun, So-Hyun Paek

**Affiliations:** Department of Emergency Medicine, CHA Bundang Medical Center, CHA University School of Medicine, Seongnam 13496, Republic of Korea; shyped85@chamc.co.kr (H.-Y.S.); suas11@chamc.co.kr (S.H.P.); mjtear@naver.com (M.-J.K.); byunyoun84@chamc.co.kr (Y.-H.B.)

**Keywords:** croup, child, pediatricians, emergency medicine, emergency medical services

## Abstract

**Background/Objectives**: With the advent of the field of pediatric emergency medicine, studies on the differences in treatment patterns between pediatricians and emergency medicine (EM) physicians in various pediatric conditions have been accumulating. This study aimed to compare croup (acute laryngotracheobronchitis) management patterns between pediatricians and EM physicians to enhance pediatric emergency care and inform the training of future specialists. **Methods**: A retrospective review of medical records was conducted for 1676 previously healthy children diagnosed with croup who visited a single pediatric emergency department (PED) of a tertiary university-affiliated hospital in South Korea, from March 2019 to February 2023. Patient characteristics, management patterns, and the impact of physician specialty on emergency care were analyzed. **Results**: EM physicians used injected dexamethasone monotherapy in 30.54% of the cases, more frequently than the 3.57% among pediatricians. In contrast, pediatricians used a combination of nebulized epinephrine and dexamethasone in 88.29% of the cases, compared with 67.71% for EM physicians. The appropriate use of nebulized epinephrine based on the Westley Croup Score was significantly higher in the EM physician group (77.64% vs. 57.89%, *p* < 0.001). Pediatricians also prescribed oral antibiotics and corticosteroids more frequently (25.13% vs. 3.13% and 81.54% vs. 22.69%, respectively; *p* < 0.001 for both). Despite these differences, there were no significant disparities in PED length of stay or 48 h revisit rates. **Conclusions**: EM physicians adhered more closely to currently accepted management algorithms for croup management. These findings underscore the need for standardized, evidence-based pediatric emergency care and provide valuable insights for training programs in this field.

## 1. Introduction

Emergency medicine (EM) is a comprehensive field that treats patients of all ages presenting to the emergency department [1]. In the early 1980s, the American Academy of Pediatrics and the American College of Emergency Physicians jointly recognized pediatric emergency medicine (PEM) as a new subspecialty, and fellowship programs began to be established in the United States [2]. In the Republic of Korea (South Korea), from the early stages of EM to the recent past, trauma and surgical conditions in children have typically been managed by EM physicians, whereas medical conditions have generally been handled by pediatricians [3]. However, with the decreasing availability of pediatric residents and the evolving need for PEM specialists, South Korea established the Korean Society of Pediatric Emergency Medicine in 2014, recognizing PEM as a vital subspecialty and fostering its advancement [4,5].

In most countries, physicians who become PEM specialists have training backgrounds in either pediatrics or EM [2]. Research has been conducted in other countries on the differences in management patterns between pediatricians and EM physicians for various pediatric conditions [6,7,8]. However, studies comparing pediatric management patterns by physician specialty are less common in East Asia compared with Western countries. In Taiwan, there were comparative studies on acute bronchiolitis, febrile children, and non-traumatic diseases between the two specialties, but no comparative studies on croup have been conducted so far in East Asia [9,10,11].

Croup (acute laryngotracheobronchitis) is one of the most common causes of acute upper airway obstruction in children aged 6 months to 6 years. It is characterized by barking cough, stridor, and hoarseness, primarily caused by a viral infection and the subsequent narrowing of the upper airway [12]. Croup is a condition of particular interest in PEM for several reasons. First, its symptoms often worsen at night [13]. Second, it may require active monitoring and airway management in the emergency department because of the potential for deterioration [14].

This study aimed to analyze the differences in croup management patterns between specialists with different training backgrounds. Our goal was to explore ways to enhance the quality of pediatric emergency care and investigate potential training improvements for EM residents and PEM fellows, particularly given the increasing number of pediatric cases being managed by EM physicians.

## 2. Materials and Methods

### 2.1. Study Design and Setting

This study was conducted through a retrospective analysis of electronic medical records (EMR), targeting previously healthy children under the age of 15 who visited the pediatric emergency department (PED) of CHA Bundang Medical Center (CHABMC) in Seongnam, South Korea, with a diagnosis of croup between 1 March 2019 and 28 February 2023. CHABMC is a tertiary teaching hospital affiliated with CHA University, located in Gyeonggi province (population of 13.66 million; area of 10,199 square kilometers), South Korea. Its PED is one of the 11 Pediatric Emergency Centers designated by the Ministry of Health and Welfare of South Korea, accepting approximately 20,000 pediatric patients under the age of 15 each year. Until February 2019, the PED was solely managed by pediatricians, with trauma cases being treated in the adult emergency department. Beginning in March 2019, its primary staffing gradually transitioned from pediatricians to EM physicians, providing a unique opportunity to compare the management patterns of physicians from different specialties. The Institutional Review Board (CHAMC 2023-12-014 and date of approval 28 December 2023) approved this study, and the requirement to obtain informed consent from the patients was waived.

### 2.2. Selection of Patients

**Figure 1:**
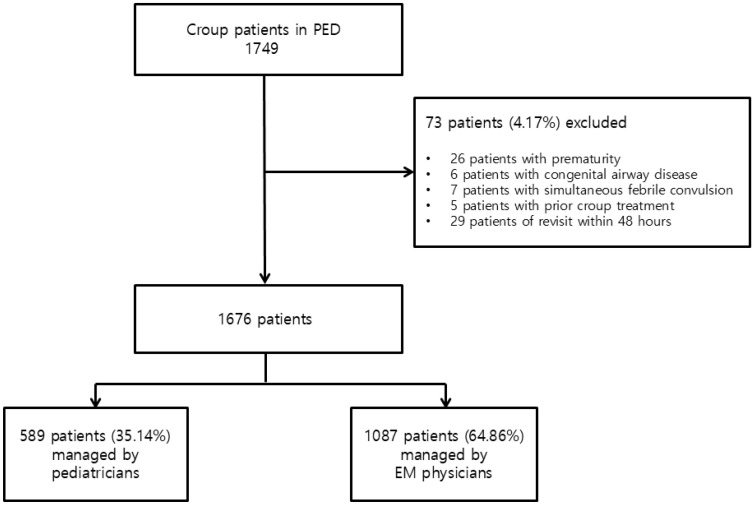
Patient flowchart. PED, pediatric emergency department; EM, emergency medicine.

Information on croup patients under the age of 15 who visited the PED during the aforementioned 4-year period was collected, and the diagnosis was categorized according to the International Classification of Diseases Version 10 (ICD-10) code for croup. Children with a history of prematurity (gestational age under 37 weeks), congenital airway disorders, and croup accompanied by febrile convulsions were excluded from this study. Croup patients who had received treatment at another medical facility before visiting our PED were also excluded. In the case of patients with revisits, data from the second visit were excluded (Figure 1).

### 2.3. Data Collection

During the specified period, the authors directly reviewed the EMR of all eligible patients. The information collected included the patient’s age (in years), sex, arrival season, arrival time, route of arrival, the Korean Triage and Acuity Scale (KTAS) level at triage, the Westley Croup Score (WCS) at triage, chief complaints, body temperature and peripheral capillary oxygen saturation (SpO2) at triage, and blood tests, including white blood cell (WBC) counts and C-reactive protein (CRP) levels. The KTAS is a tool used in South Korea to evaluate the severity of patients presenting to the emergency department, with lower numbers indicating higher severity [15]. The seasons were classified according to the usual seasonal divisions in East Asia: spring (March to May), summer (June to August), autumn (September to November), and winter (December to February) [16]. The arrival time was categorized as day (8 a.m. to 4 p.m.), evening (4 p.m. to midnight), and night (midnight to 8 a.m. the next day) [17].

Our study compared whether plain radiographs were taken, the types of medication administered, the use of nebulized epinephrine, the inclusion of antibiotics or corticosteroids in discharge medications, and the disposition of patients managed by pediatricians and those managed by EM physicians. We also evaluated the appropriateness of nebulized epinephrine use, specifically whether it was administered to croup patients with a WCS higher than 2.

Additionally, comparisons were made between the two groups regarding PED length of stay (PEDLOS) and revisit rates within 48 h. A “pediatrician” was defined as a physician who had completed a 1-year internship and a 4-year pediatric residency to become board-certified. An “EM physician” was defined as a physician who had completed an internship and an emergency medicine residency within the same timeframe to obtain board certification. The specialists included in this study, both pediatricians and EM physicians, started working in the PED following one to two years of fellowship training.

The primary outcomes included differences in management patterns and the impact on the emergency care of croup patients, according to physician specialties. The secondary outcomes addressed the seasonal and annual trends in the number of croup patients, as well as the proportions of viruses causing croup identified through reverse transcription-polymerase chain reaction (RT-PCR) analysis during the study period, including severe acute respiratory syndrome coronavirus 2 (SARS-CoV-2).

### 2.4. Data Analysis

Statistical analyses were conducted using SAS version 9.4 (SAS Institute Inc., Cary, NC, USA). Descriptive statistics are reported as medians with the first and the third quartiles (Q1 and Q3) rounded to two decimal places. Categorical variables are presented as frequencies and percentages. The statistical methods included the Chi-square test, the Wilcoxon rank sum test, and Fisher’s exact test. Statistical significance was defined as a *p*-value of less than 0.05.

## 3. Results

A total of 1749 children under the age of 15 who visited our PED from 1 March 2019 to 28 February 2023, with a diagnosis of croup, were enrolled in this study. Seventy-three individuals were excluded based on the stated data collection criteria, leaving a total of 1676 patients in the study. Of these, 589 were managed by pediatricians and 1087 by EM physicians (Figure 1). During the relevant period, eight pediatricians and eight EM physicians worked in the PED. Patients who were seen by a specialist from the outset naturally received treatment based on the specialist’s judgment. Similarly, for those initially examined by pediatric or EM residents, since the specialist was involved in all tests and treatments, it can be assumed that key decisions, such as treatment plans and patient management, were ultimately made according to the specialist’s intention.

### 3.1. Demographic Characteristics of Participants

Table 1 shows the baseline characteristics of the final patient cohort included in this study, divided into a group managed by pediatricians (pediatrician group) and another group managed by EM physicians (EM physician group) for all items. In both groups, the median age was 2 years, and the proportion of males was higher than females (65.39%). Regarding the arrival season to the PED, the pediatrician group had the highest number of patients in autumn (29.54%), whereas the EM physician group had the highest number in winter (36.34%), showing a significant difference. In terms of the arrival time, the largest number of croup patients in both groups presented during the night (i.e., from midnight to 8 a.m.), although a statistically significant difference was seen in the specific proportions. Most patients (94.27%) visited the PED directly as walk-ins.

The median KTAS level at triage was 3 in both groups. When the KTAS levels of 1–3 were classified as emergencies and 4–5 as non-emergencies, the proportion of emergency cases in the pediatrician group was 88.29%, which was statistically significantly higher than 84.54% in the EM physician group. The most common chief complaint in both groups was barking cough (73.57%).

The median body temperature of the croup patients was 37.7 °C in both groups, with no significant difference observed. Similarly, the SpO2 levels of patients were not significantly different between the two groups. The results of the laboratory tests, which included WBC counts and CRP levels, also showed no statistically significant differences.

### 3.2. Comparison of Management Patterns by Physician Specialties

The differences in management patterns between both groups are summarized in Table 2. The WCS was a median of 3 in the pediatrician group and a median of 2 in the EM physician group, which were statistically significantly different. Plain radiographs of the neck and chest were performed in over 99% of the patients in both groups.

In terms of management in the PED, the use of injected dexamethasone alone was significantly more common among EM physicians (30.54%) compared with pediatricians (3.57%). Pediatricians used nebulized epinephrine alone in 5.94% of the cases and combined it with injected dexamethasone in 88.29% of the cases. In contrast, EM physicians used nebulized epinephrine alone in only 0.37% of the cases and combined it with injected dexamethasone in 67.71% of the cases, which was lower in both categories.

Among all croup patients, 95.94% received intramuscular or intravenous (IV) injections of dexamethasone, with the EM physician group administering it approximately 6% more frequently than the pediatrician group. The median dose of injected dexamethasone was significantly higher in the EM physician group (0.3 mg/kg) than in the pediatrician group (0.26 mg/kg).

In all croup patients included in this study, laboratory tests were always performed alongside IV fluid therapy. The proportion of such patients was higher in the EM physician group (19.65%) than in the pediatrician group (13.61%). The rate of appropriate nebulized epinephrine use, according to the WCS, was significantly higher in the EM physician group, at 77.64%, compared with 57.89% in the pediatrician group. The proportion of patients who received oxygen via nasal cannula, high-flow nasal cannula, or mechanical ventilation was not significantly different between the two groups.

The prescription of antibiotics at discharge was notably higher in the pediatrician group, with 25.13%, compared with 3.13% in the EM physician group, for a difference of 22.6%. Similarly, corticosteroids were prescribed by pediatricians at discharge in 81.54% of the cases, which was 58.85% higher than the 22.69% observed in the EM physician group, revealing a significant difference.

### 3.3. The Impact of Physician Specialty on the Emergency Care of Croup Patients

Table 3 presents several comparisons of the impact of emergency care on the croup patients by physician specialty. EM physicians admitted 11.69% of the croup patients who visited the PED to the general ward, which was significantly higher than the 7.13% admitted by pediatricians. There were no intensive care unit (ICU) admissions in the pediatrician group, whereas the EM physician group had four cases (0.37%). Both specialties showed improvements in croup patient outcomes, with 92.87% of the children treated by pediatricians and 87.94% treated by EM physicians improving and being discharged after treatment in the PED. The median PEDLOS was also similar, with no significant difference (86 min for pediatricians and 97 min for EM physicians). The revisit rates within 48 h were 2.55% for pediatricians and 1.29% for EM physicians, with pediatrician’s care resulting in a 1.26% higher rate; however, this difference was not statistically significant. No mortality was reported for any croup patient seen during this period.

### 3.4. Trends in Croup Patients by Year and Season

**Figure 2:**
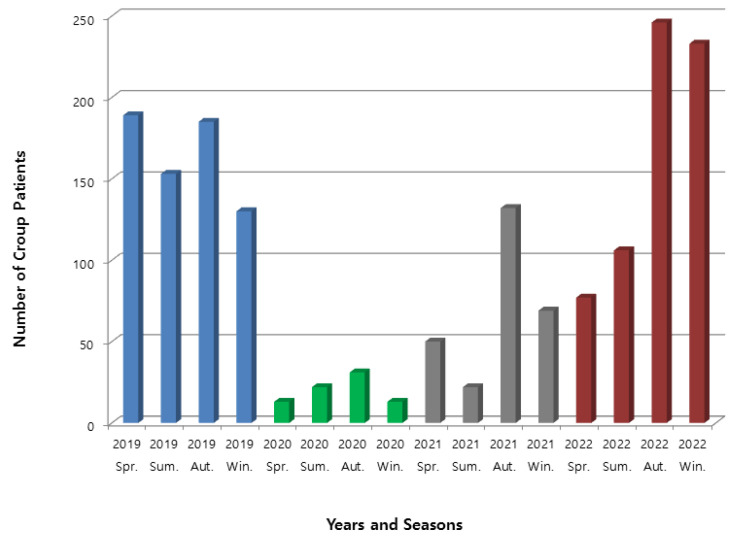
The trend in the number of croup patients visiting the PED by year and season. Spr., spring; Sum., summer; Aut., autumn; Win., winter.

This study identified a trend in the number of croup patients over a 4-year period, annually and by season, as depicted in Figure 2. The number of croup patients, which ranged from 130 to 190 per season, declined sharply starting in spring 2020 and remained below 50 cases per season until before autumn 2021. After spring 2022, the number of croup patients began to increase again, and by autumn 2022, it had recovered to pre-SARS-CoV-2 levels.

At our PED, during the specified period, respiratory virus RT-PCR tests were conducted on 194 croup patients with parental consent. A total of 219 positive results were identified in 171 of these patients. Throughout the entire period, the highest proportion was parainfluenza virus at 24.2%, followed by influenza virus (22.37%), enterovirus (12.33%), rhinovirus (12.33%), SARS-CoV-2 (8.68%), and respiratory syncytial virus (7.76%).

## 4. Discussion

In the present study, we analyzed the baseline characteristics of croup patients, differences in management patterns by physician specialties, and trends in the number of patients over the 4-year period from March 2019 to February 2023 at a single PED.

### 4.1. Analysis of Differences in Management Patterns by Specialties

The main focus of this study was to analyze the differences in management patterns between specialists with two different training backgrounds when managing croup patients (Table 2). The median WCS difference between the pediatrician group and the EM physician group was 1. However, considering that the WCS inevitably has a subjective aspect, this small difference does not necessarily indicate a distinct difference. Despite the one-point difference, the admission rate to the general ward was higher in the EM physician group, and four ICU cases were seen only in the EM physician group. We consider it more important to evaluate how management was influenced by the scores.

Based on previous studies, there is no single, universally accepted guideline for croup treatment internationally, and there are minor differences depending on the institution [18]. However, widely accepted algorithms do exist, which share common views, at least on the use of corticosteroids, nebulized epinephrine, and the unnecessary use of additional antibiotics or corticosteroids as discharge medications [19].

The mean dose of injected dexamethasone was higher in the EM physician group than in the pediatrician group. However, previous studies reported no significant differences in emergency department return visits, (re)admission, length of hospital stay, or intubation risk among those who received 0.15 mg/kg, 0.3 mg/kg, or 0.6 mg/kg dexamethasone [20]. Therefore, we could not determine the superiority between the two specialties based on dexamethasone doses.

The proportion of IV fluid therapy and concurrent laboratory tests was significantly higher in the EM physician group. This may have been because our EM orderset recommends laboratory tests in cases of fever lasting more than 72 h, indicating that EM physicians tended to follow the orderset in their management. However, in cases of clinically evident croup, there is no clear evidence that IV fluid therapy and laboratory tests are mandatory unless hospitalization is required or signs of deterioration in the general condition are present [21].

### 4.2. Adherence to Currently Accepted Management Algorithms by Physician Specialties

In terms of management combinations, administering only injected dexamethasone was significantly more common in the EM physician group than in the pediatrician group. The currently accepted algorithms recommend using corticosteroid monotherapy for mild croup (WCS 1–2) and advise the use of nebulized epinephrine starting from moderate croup (WCS 3–7) [22]. Based on this analysis, the EM physician group used medication more in accordance with the currently accepted algorithms than the pediatrician group.

As a result of proactive vaccination against *Corynebacterium diphtheriae* and *Haemophilus influenzae* type b, croup is now largely believed to be caused by viruses [23]. This study observed that the EM physician group managed the condition more in accordance with currently accepted algorithms than the pediatrician group, as they used antibiotics less frequently. The findings suggest that if concomitant pneumonia is present on chest radiographs, as in parainfluenza virus type 3, the pediatricians might have a greater likelihood of prescribing oral antibiotics. However, further research is needed to explore this possibility.

Corticosteroids, most commonly dexamethasone, are the mainstay treatment for all croup patients, and a single dose administered in the hospital is generally considered sufficient [20]. In this study, the pediatrician group prescribed additional oral corticosteroids as discharge medication after corticosteroid administration in the PED more frequently than the EM group. This contrasts with a previous study in the United States, which found no significant differences in antibiotic and corticosteroid discharge prescriptions for croup patients among pediatricians, general EM physicians, and PEM specialists [7]. The reason can be speculated from two perspectives. First, it may be related to the relatively lower injected dose of dexamethasone in the pediatrician group, as pediatricians might have determined that continued corticosteroid administration was necessary after discharge. Second, EM physicians may have had a stronger tendency to follow ordersets, which are generally based on a standardized approach, compared with pediatricians.

### 4.3. The Impact of Physician Specialties in the Pediatric Emergency Care

Several previous studies investigated whether differences in medical specialties had a significant impact on the treatment outcomes of pediatric patients in the emergency department. A study conducted in the United States found no significant differences in admission rates among croup patients treated by pediatricians, general EM physicians, and PEM specialists. However, the length of stay in the emergency department was highest in the general EM physician group [7]. In a study in Canada, children with various illnesses who visited the emergency department had a lower admission rate when treated by a PEM specialist compared with general pediatricians and general EM physicians [24]. In our study, EM physicians showed a higher rate of general ward admissions compared with pediatricians; however, since our study was limited to patients with croup, a direct comparison was difficult. Research on this topic is insufficient in East Asia, with only three studies from Taiwan examining variations in management by specialty [9,10,11]. Therefore, further studies in this area are needed.

### 4.4. Demographic Characteristics and Trends in Croup Patients

In both groups, the highest number of croup patients visited the PED during the night, which aligns with the established finding that croup symptoms typically worsen at night [13]. Among all croup patients, the number of boys was approximately twice that of girls, a pattern also confirmed by previous studies [25]. Although existing studies have indicated that the oral route is preferred for administering corticosteroids to children with mild croup, our PED predominantly used intramuscular or IV injections [26]. We assume that the injectable forms were preferred because many croup patients exhibited irritability at the time of presentation, and since there is no dexamethasone syrup formulation for children in South Korea, unpalatable powdered medicine is the oral alternative.

We also observed trends among croup patients visiting our PED over a 4-year period. From early 2020, when the SARS-CoV-2 pandemic began, the number of croup patients sharply declined but started to rise again in the second half of 2021 and showed a significant increase starting in autumn 2022. In April 2022, South Korea lifted all social distancing measures, including mask-wearing and restrictions on social gatherings [27]. Since then, the number of croup patients has been trending upward, reaching levels similar to those before the SARS-CoV-2 pandemic (Figure 2). The RT-PCR analysis of respiratory viruses in croup patients showed that SARS-CoV-2 became one of the major causative viruses.

### 4.5. The Importance of Clinical Practice Standardization in This Field

The characteristics of emergency care, including the need to quickly determine the assessment, management, and disposition, coordination with various departments, and the fact that different physicians work during different shifts, emphasize the extreme importance of evidence-based clinical practice standardization. Similarly, in pediatric emergency care, training residents and PEM fellows to diagnose, treat, and determine the disposition of patients according to the latest clinical guidelines, as well as establishing systems, such as ordersets or clinical pathways, are essential. This is particularly important in countries like South Korea, which are still developing their PEM programs. A past study on pediatric residents showed that teaching a standardized management algorithm for croup at the beginning of emergency department rotations led to educational outcomes, such as a decrease in the use of nebulized epinephrine for mild croup, the reduced use of plain radiographs, and fewer antibiotic prescriptions [28]. A similar improvement was observed in another study where the croup clinical pathway was adopted, leading to a significant reduction in PEDLOS and admission rates [29]. In our PED, the EM orderset specifies croup management based on the WCS, which likely contributed to these improvements. In this study, we did not find significant differences in either the PEDLOS or the revisit rate to the PED within 48 h between the two groups (Table 3). However, we expect that continuous efforts toward the standardization of clinical practice will improve the quality of care and education in the future, based on past research findings [30].

This study had the following limitations. This was a retrospective study based on EMR reviews. However, in studies that compare management patterns, conducting a prospective study could influence physicians’ treatment methods (i.e., the Hawthorne effect). Thus, being retrospective is not necessarily a drawback in this type of study. This study was conducted at a single PED, which restricts the generalizability of the findings and may only reflect prevalence in the local community, differing from national data. Additionally, the number of croup patients visiting the PED and the composition of causative viruses has changed since the SARS-CoV-2 pandemic in mid-2020, making it difficult to assume that the patient population has uniform characteristics.

## 5. Conclusions

This pioneering study in East Asia aimed to compare the management patterns of croup patients between pediatricians and EM physicians to gather insight into improving pediatric emergency care. Despite some variations in management, no significant difference was found between the two groups in terms of PEDLOS and the PED revisit rate within 48 h. EM physicians tended to adhere more closely to currently recommended algorithms, including lower antibiotic usage and the appropriate use of nebulized epinephrine based on croup severity. This study not only provides valuable insight for EM physicians and residents treating pediatric patients but also highlights the importance of the evidence-based standardization of clinical practice, encouraging further research in this field.

## Figures and Tables

**Table 1:** Baseline characteristics of patients.

	Total	Pediatricians (n = 589)	EM Physicians (n = 1087)	*p*-Value
Age (year), median (Q1, Q3)	2 (1, 3)	2 (1, 3)	2 (1, 3)	0.776 *
Male sex, N (%)	1096 (65.39)	380 (64.52)	716 (65.87)	0.578 †
Arrival season				
Spring (Mar.–May)	329 (19.63)	168 (28.52)	161 (14.81)	<0.001 †
Summer (Jun.–Aug.)	303 (18.08)	119 (20.2)	184 (16.93)	
Autumn (Sep.–Nov.)	521 (31.09)	174 (29.54)	347 (31.92)	
Winter (Dec.–Feb.)	523 (31.21)	128 (21.73)	395 (36.34)	
Arrival time				
Day (08:00–16:00)	247 (14.74)	70 (11.88)	177 (16.28)	0.022 †
Evening (16:00–MN)	558 (33.29)	191 (32.43)	367 (33.76)	
Night (MN–08:00)	871 (51.97)	328 (55.69)	543 (49.95)	
Route of arrival				
Direct	1580 (94.27)	561 (95.25)	1019 (93.74)	0.381 **
EMS	93 (5.55)	27 (4.58)	66 (6.07)	
Transfer	3 (0.18)	1 (0.17)	2 (0.18)	
KTAS, median (Q1, Q3)	3 (3, 3)	3 (3, 3)	3 (3, 3)	0.070 *
KTAS 1–3, N (%)	1439 (85.86)	520 (88.29)	919 (84.54)	0.036 †
KTAS 4–5, N (%)	237 (14.14)	69 (11.71)	168 (15.46)	
Chief complaint				
Barking cough	1233 (73.57)	426 (72.33)	807 (74.24)	0.228 †
Dyspnea	173 (10.32)	57 (9.68)	116 (10.67)	
Fever	230 (13.72)	94 (15.96)	136 (12.51)	
Etc.	40 (2.39)	12 (2.04)	28 (2.58)	
Vital sign				
BT (°C), median (Q1, Q3)	37.7 (36.9, 38.4)	37.7 (36.9, 38.4)	37.7 (36.9, 38.4)	0.828 *
SpO2 (%), median (Q1, Q3)	100 (98, 100)	100 (99, 100)	100 (98, 100)	0.711 *
Laboratory results, median (Q1, Q3)				
WBC (/µL)	10,700 (8050, 13,960)	10,065 (8060, 13,250)	10,820 (8050, 14,230)	0.525 *
CRP (mg/dL)	0.47 (0.16, 1.18)	0.53 (0.21, 1.19)	0.45 (0.15, 1.18)	0.381 *

* *p*-value by Wilcoxon rank sum test; ** *p*-value by Fisher’s exact test; † *p*-value by Chi-square test; EM, emergency medicine; Q1, first quartile; Q3, third quartile; Mar., March; Jun., June; Aug., August; Sep., September; Nov., November; Dec., December; Feb., February; MN, midnight; EMS, emergency medical service; KTAS, Korean Triage and Acuity Scale; BT, body temperature; SpO2, peripheral capillary oxygen saturation; WBC, white blood cell; CRP, C-reactive protein.

**Table 2:** Comparison of management patterns of pediatricians and EM physicians.

	Total	Pediatricians (n = 589)	EM Physicians (n = 1087)	*p*-Value
Westley croup score, median (Q1, Q3)	2 (1, 4)	3 (2, 3)	2 (1, 4)	<0.001 *
Plain radiograph of neck and chest	1674 (99.88)	589 (100)	1085 (99.82)	0.544 **
Management				
Injected dexamethasone only	353 (21.06)	21 (3.57)	332 (30.54)	<0.001 †
Injected dexamethasone and nebulized epinephrine	1256 (74.94)	520 (88.29)	736 (67.71)	
Nebulized epinephrine only	39 (2.33)	35 (5.94)	4 (0.37)	
No dexamethasone or epinephrine	28 (1.67)	13 (2.21)	15 (1.38)	
Injected dexamethasone use				
Used	1608 (95.94)	542 (92.02)	1066 (98.07)	<0.001 †
Not used	68 (4.06)	47 (7.98)	21 (1.93)	
Injected dexamethasone dose (mg/kg), median (Q1, Q3)	0.29 (0.26, 0.3)	0.26 (0.18, 0.29)	0.3 (0.28, 0.3)	<0.001 *
IV fluid therapy and laboratory test	293 (17.52)	80 (13.61)	213 (19.65)	0.002 †
Appropriate use of nebulized epinephrine	1185 (70.7)	341 (57.89)	844 (77.64)	<0.001 †
Oxygen supplement	29 (1.73)	12 (2.04)	17 (1.56)	0.478 †
Prescription of oral antibiotics at discharge	182 (10.86)	148 (25.13)	34 (3.13)	<0.001 †
Prescription of oral corticosteroids at discharge	662 (44.16)	446 (81.54)	216 (22.69)	<0.001 †

* *p*-value by Wilcoxon rank sum test; ** *p*-value by Fisher’s exact test; † *p*-value by Chi-square test; EM, emergency medicine; Q1, first quartile; Q3, third quartile; IV, intravenous.

**Table 3:** The impact of specialty on the emergency care of croup patients.

	Total	Pediatricians (n = 589)	EM Physicians (n = 1087)	*p*-Value
Disposition				
Discharge	1502 (89.67)	547 (92.87)	955 (87.94)	0.002 **
Admission to GW	169 (10.09)	42 (7.13)	127 (11.69)	
Admission to ICU	4 (0.24)	0 (0)	4 (0.37)	
PEDLOS (min), median (Q1, Q3)	93 (57, 157)	86 (60, 138)	97 (54, 170)	0.138 *
Revisit (N, %)	29 (1.73)	15 (2.55)	14 (1.29)	0.059 †

* *p*-value by Wilcoxon rank sum test; ** *p*-value by Fisher’s exact test; † *p*-value by Chi-square test; EM, emergency medicine; GW, general ward; ICU, intensive care unit; PEDLOS, pediatric emergency department length of stay; Q1, first quartile; Q3, third quartile.

## Data Availability

The data presented in this study are available upon request from the corresponding author. The data are not publicly available because of restrictions imposed by the hospital review, which mandates the destruction of the data within 3 years.
